# Reply to “Why Secondary Analyses in Vitamin D Clinical Trials Are important and How to Improve Vitamin D Clinical Trial Outcome Analyses—A Comment on “Extra-Skeletal Effects of Vitamin D”, *Nutrients* 2019, *11*, 1460”

**DOI:** 10.3390/nu11092188

**Published:** 2019-09-11

**Authors:** Madhusmita Misra, Rose Marino

**Affiliations:** Division of Pediatric Endocrinology, Massachusetts General Hospital and Harvard Medical School, Boston, MA 02114, USA

We appreciate the interest of Drs. Grant and Boucher [1] in our recent review “Extra-Skeletal Effects of Vitamin D” [2]. They specifically comment on our interpretation of the findings of two studies recently published in the *New England Journal of Medicine* that were included in our review [3,4], and discuss results of secondary and post-hoc analyses from these studies. They conclude by advocating for the analysis of results of future studies based on serum 25-hydroxy vitamin D (25(OH)D) concentrations and the importance of highlighting data from prespecified secondary analyses. 

Drs. Grant and Boucher are correct in that we did indeed go by the primary intent-to-treat analysis reported in the *New England Journal of Medicine* for both studies. 

In the paper by Manson et al. examining effects of vitamin D supplementation on prevention of cancer and cardiovascular disease in men 50 years or older and in women 55 years or older [3], the study found no impact of vitamin D supplementation on cancer or cardiovascular disease prevention on primary analysis, although on subgroup analysis, the group with a BMI of <25 kg/m^2^ did appear to have better outcomes for cancer prevention than those with higher BMI. The other impact was on reducing the rate of death from cancer on a test of proportionality over time, a secondary study endpoint. Of note, in the Statistical Methods, the authors indicate the following for secondary and exploratory endpoint testing: “However, there was no control for multiple hypothesis testing, and no formal adjustment was made to the *P* values or confidence intervals. Thus, results regarding secondary and exploratory endpoints, as well as those regarding subgroups, should be interpreted with caution”.

In the paper by Pittas et al. examining effects of vitamin D supplementation on prevention of type 2 diabetes in participants meeting two out of three criteria for prediabetes [4], the authors report that “Results of the subgroup analyses were consistent with the findings of the main analysis; there was no apparent heterogeneity of treatment effect across the prespecified subgroups”. A figure representing hazard ratios for subgroups does suggest a beneficial effect in the group with a BMI of <30 kg/m^2^. This merits further study, although the risk for type 2 diabetes is typically higher in those with obesity, and thus one would have liked to see better results in the higher BMI group. In the subgroup analysis, results did not differ for subgroups with 25(OH)D levels of <20 vs. ≥20 ng/ml. However, a post-hoc analysis suggested a beneficial effect of vitamin D supplementation in the group with levels of <12 ng/ml. The authors indicate in the Statistical Methods that “‘No adjustments were made for the …… post hoc analyses for the primary outcome; therefore, only point estimates and 95% confidence intervals are presented without *P* values”. 

We agree with Drs. Grant and Boucher that achieving optimal 25(OH)D levels is more difficult in those with a higher BMI, and that it may be more important to target a range of 25(OH)D levels with vitamin D supplementation in future studies than to go with a fixed dose of vitamin D. However, we were unable to find information in the papers regarding 25(OH)D levels attained within subgroups (and not just randomization groups) following supplementation. 

For all these reasons, for both studies, we preferred to report the results of the primary study outcomes, as indicated by the study authors. Future studies are necessary to determine the impact of targeting a range of 25(OH)D levels for interventional studies of vitamin D supplementation. 
